# Supplementary figures and images for: Regular Exercise and Weight-Control Behavior Are Protective Factors against Osteoporosis for General Population: A Propensity Score-Matched Analysis from Taiwan Biobank Participants
Source: Nutrients. 2022 Feb 2;14(3):641. doi: 10.3390/nu14030641 (PMC8838409; doi:10.3390/nu14030641)

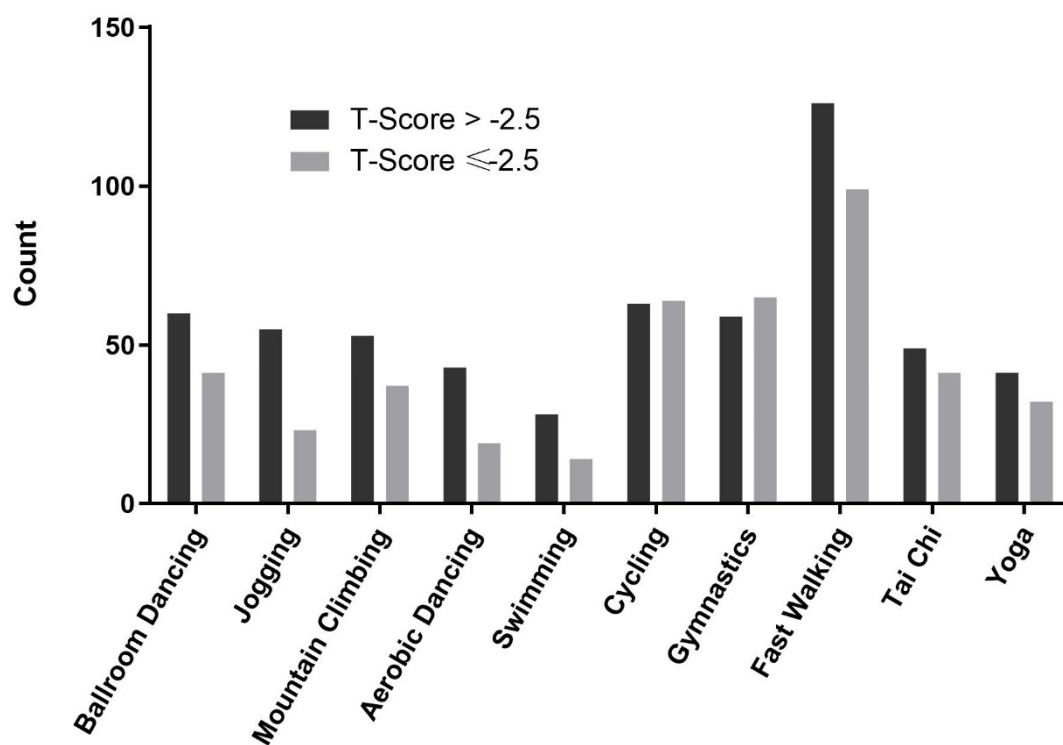

Figure S1. Count of different exercises (maximum of three exercises by each participant).

Supplement: Supplementary file 1 [file nutrients-14-00641-s001.zip › nutrients-1528503-supplementary.pdf]
